# Editorial: Sport activity: From beneficial effects to cardiac disease

**DOI:** 10.3389/fphys.2022.1094048

**Published:** 2022-11-22

**Authors:** Davide Romagnolo, Andre D′Avila, Federico Migliore, Pasquale Vergara

**Affiliations:** ^1^ IRCCS San Raffaele Hospital, Vita-Salute San Raffaele University, Milan, Italy; ^2^ Beth Israel Deaconess Medical Center, Harvard Medical School, Harvard-Thorndike Electrophysiology Institute, Boston, MA, United States; ^3^ Department of Cardiac, Thoracic and Vascular Sciences and Public Health, University of Padova, Padova, Italy; ^4^ Cardiac Electrophysiology and Arrhythmology Unit, IRCCS San Raffaele Hospital, Milan, Italy

**Keywords:** sport, cardiology, exercise, cardiomyopathies, arrhythmias

This editorial presents a summary of the articles published in Sport Activity: From Beneficial Effects to Cardiac Disease. The aim of this Research Topic is to encourage studies investigating the complex relationship between physical activity and cardiovascular health. In particular, the publications selected in this collection highlights how sport activity may have opposite effects on cardiovascular system, ranging from beneficial effects to cardiac disease. Indeed, although beneficial effects of sport activity are well-known and have been extensively studied (Sharma et al., 2015), the subtended physiological mechanisms have not been completely clarified (Hawley et al., 2014). In addition, only in recent years the “dark side” of athletic training has emerged, ranging from morpho-functional adaptations overlapping with cardiomyopathies to myocardial injury and arrythmias such as atrial fibrillation, sinus node disease and atrioventricular blocks (Sharma et al., 2015). The following are the articles in Sport Activity: From Beneficial Effects to Cardiac Disease.

The review by Aminuddin et al. highlights the inverse correlation between muscle function and arterial stiffness (AS) both in healthy adults and patients affected from cardiovascular disease (CVD) or with CVD risk factors, as proved by 17 original research articles published since 1971. AS increases with aging and arterial hypertension, mainly due to a low-grade proinflammatory status occurring in arterial walls, inducing endothelial dysfunction (Wang et al., 2018). Intuitively, impaired vascular function may limit the increase in blood supply to skeletal muscles during exercise, hampering muscular growth and favoring frailty (Jeon et al., 2021). Aerobic exercise is known to induce a favorable vascular adaptation, improving AS, and this effect is proportional to aerobic exercise intensity and baseline AS (Ashor et al., 2014). This phenomenon is mainly mediated by anti-oxidant effects and increased vasodilation mediated by endothelial production of nitric oxide (NO) induced by exercise (Ashor et al., 2014). In this way, aerobic sport promotes anti-aging effects on cardiovascular system.

Still concerning to exercise effects on skeletal muscle, Nyberg et al. in their original research showed how progressive increase in resistance training volume may alter fiber-type distribution and oxidative muscle protein levels in quadriceps of patients affected from chronic obstructive pulmonary disease (COPD). In particular, they describe an increased proportion of Type I fibers and mitochondrial transcription factor A (TfAM) muscle protein levels, as well as a decreased proportion of Type II fibers in patients who showed increasing in resistance training volume compared to patients who did not. Of note, TfAM overexpression may be involved in reverse remodeling after myocardial infarction (Ikeuchi et al., 2005), and it has been extensively demonstrated a reduction in type I fibers in patient affected from heart failure (Adams et al., 2017), ultimately contributing to exercise intolerance in a vicious circle.

Understanding the molecular mechanisms of beneficial effects of aerobic sport on cardiovascular health is of great interest and may have relevant implications on clinical practice. Lv et al. reviewed the role of mitochondria-associated endoplastmatic reticulum membranes (MAMs) in calcium homeostasis, lipid homeostasis, apoptosis, and inflammation. MAMs are composed by proteins, the expression of which may be altered by exercise, explaining the cardioprotective effects of exercise preconditioning. Therefore, studying exercise patterns directly influencing MAMs composition may help increase efficacy of preventive and rehabilitative cardiology.

Beyond cardioprotection, aerobic exercise may also provide a therapeutic solution to already developed cardiac diseases. Wang et al. demonstrated an interesting correlation between aerobic exercise and reverse cardiac remodeling in a murine model of diabetic cardiomyopathy. This effect appears to be due, at least in part, to P2X7 purinergic receptors (P2X7R) downregulation induced by treadmill exercise, resulting in reduced fibrotic and hypertrophic markers, and apoptosis-related proteins as well. Similar benefits are present in P2X7R knock out diabetic mice, underlying the pathophysiological role of this molecule.

Translating these finding to clinical practice, Zhao et al. compared echocardiographic myocardial work with aerobic capacity measured at cardiopulmonary exercise test in pre-adolescent male basketball players. Pressure-strain loop analysis derived from arterial pressure and two-dimensional speckle-tracking echocardiography (STE) provides a novel, non-invasive and less load-dependent method for evaluation of left ventricular systolic function. Zhao et al. found out an interesting correlation of global work index (GWI) and global work energy (GWE), both indexed to body surface area (BSA), with VO_2_max and peak O_2_ pulse. This study suggests further applications of STE–derived myocardial work in prediction of left ventricular contractile reserve and exercise tolerance, as proposed by other authors (Edwards et al., 2021; Su et al., 2022).

Finally, Maffetone and Laursen remind us that despite benefits listed above, athletic training may have drawbacks. Indeed, athletes have a 2.4-to-4.5-fold increased risk of sudden cardiac death (SCD) compared to general population, and this can not be neglected (Emery and Kovacs, 2018). The authors emphasize that additional SCD risks associated with post-COVID-19 infection and vaccination, highlighting the significant incidence of subclinical COVID-19—related myocarditis and pericarditis in healthy athletes. Catecholaminergic surge linked to overtraining may induce fatal ventricular tachyarrhythmias in patients affected from subclinical myocarditis and myopericarditis, explaining, at least in part, the increased SCD rate observed worldwide in 2021 compared to 2001–2020.

In summary, this Research Topic highlights the duality of exercise on cardiovascular health, ranging from physiological beneficial effects to pathological detrimental results, many of which are incompletely understood and still under investigation.
